# The Bacteriology of Panophthalmitis

**Published:** 1904-09-10

**Authors:** 


### THE BACTERIOLOGY OF PANOPHTHALMITIS.

There are few mischances more distressing to everyone concerned than the failure of a cataract operation, and yet, despite the utmost care, there still occurs in the practice of every surgeon an occasional instance where the eye is destroyed by panophthalmitis following extraction of the lens. That bacteriology may give useful aid in rendering such cases still less frequent than they now are is the moral of a paper by Dr. R. H. Johnston.[1] He points out that the work of several investigators has shown that Fraenkel's pneumococcus is the most frequent cause of panophthalmitis, and Dr. Johnston's own investigations are confirmatory of this fact. He made bacteriological examinations of two cases of panophthalmitis, one following cataract extraction in a patient who had recently suffered from pneumonia, and the other being consequent on accidental trauma, and in both the pneumococcus was found in large numbers. The dangers of cataract extraction when a mucocele is present are sufficiently well-known, and Dr. Johnston quotes three cases where such a combination resulted in loss of the eye from septic infection. In all of these cases the pneumococcus was found to be the cause. It has been said that this micro-organism is frequently present in mucoceles; and it is known to be the most common cause of ulcus serpens corneæ. In concluding his paper Dr. Johnston suggests that previous to an operation for cataract a bacteriological examination of the conjunctiva should be made to ascertain the absence or presence of the pneumococcus, for he believes that it will be possible, by deferring operation in the latter eventuality, to decrease the number of cases of panophthalmitis following extraction of the lens for cataract.

[1] Med. News, Aug. 20.

